# Dynamic Network of Transcription and Pathway Crosstalk to Reveal Molecular Mechanism of MGd-Treated Human Lung Cancer Cells

**DOI:** 10.1371/journal.pone.0031984

**Published:** 2012-05-31

**Authors:** Liyan Shao, Lishan Wang, Zhiyun Wei, Yuyu Xiong, Yang Wang, Kefu Tang, Yang Li, Guoyin Feng, Qinghe Xing, Lin He

**Affiliations:** 1 Bio-X Institutes, Key Laboratory for the Genetics of Developmental and Neuropsychiatric Disorders (Ministry of Education), Shanghai Jiao Tong University, Shanghai, China; 2 Institute for Nutritional Sciences, Shanghai Institutes of Biological Sciences, Chinese Academy of Sciences, Shanghai, China; 3 Institutes of Biomedical Sciences, Fudan University, Shanghai, China; University of Texas Health Science Center at San Antonio, United States of America

## Abstract

Recent research has revealed various molecular markers in lung cancer. However, the organizational principles underlying their genetic regulatory networks still await investigation. Here we performed Network Component Analysis (NCA) and Pathway Crosstalk Analysis (PCA) to construct a regulatory network in human lung cancer (A549) cells which were treated with 50 uM motexafin gadolinium (MGd), a metal cation-containing chemotherapeutic drug for 4, 12, and 24 hours. We identified a set of key TFs, known target genes for these TFs, and signaling pathways involved in regulatory networks. Our work showed that putative interactions between these TFs (such as ESR1/Sp1, E2F1/Sp1, c-MYC-ESR, Smad3/c-Myc, and NFKB1/RELA), between TFs and their target genes (such as BMP41/Est1, TSC2/Myc, APE1/Sp1/p53, RARA/HOXA1, and SP1/USF2), and between signaling pathways (such as PPAR signaling pathway and Adipocytokines signaling pathway). These results will provide insights into the regulatory mechanism of MGd-treated human lung cancer cells.

## Introduction

Lung cancer is a worldwide leading cause of cancer-related death with a 5-year survival rate of less than 15% [1]. Several molecular markers associated with lung cancer progression have been identified, including TGF, MET, TP53, HIF1A, APC, KRAS, and EGFR [2].

Transcription factors (TFs) and pathways play critical roles in etiologies of lung cancer. For example, the transcription factor E2F-1 is over-expressed in lung cancer cell, and the level is enhanced by deregulated pRb-p53-MDM2 circuitry [3]. Transcriptional regulation analysis has shown that the promoter activity and expression level of Sp1 are regulated by Ets-1 in A549 lung cancer cells. Functional analysis of two Ets-1-binding sites in Sp1 promoter suggests that only Ets-1-binding site −413 to −404 is involved in the activation of Sp1 promoter [4]. It has also been well-documented that the expression of cPLA2 is critical for the transformed growth of lung cancer and for phorbol 12-myristate 13-acetate (PMA)-activated signal transduction pathway which is involved in enzymatic activation of cPLA2. Studies reveal that c-Jun/nucleolin and c-Jun/Sp1 complexes play an important role in PMA-regulated cPLA2a gene expression [5]. In addition, several pathways involved in lung cancer progression have been demonstrated, such as PI3K/AKT pathway, TGF-beta signaling pathway, Wnt pathway, JAK/STAT pathway, and MAPK/ERK pathway [6], [7], [8], [9].

High-throughput techniques in biology, such as microarray, have generated a large amount of data that can potentially provide systems-level information regarding the underlying dynamics mechanisms [10]. To extract meaningful information (TFs and pathways information) from high-throughput expression data, we employed NCA and PCA to construct and analyze the dynamic regulatory network in MGd-treated human lung cancer cells.

NCA, developed by James Liao [10], is a network structure-driven framework for deducing regulatory signal dynamics. NCA models the expression of a gene as a linear combination of the activity of each transcription factor that controls the expression of the gene. NCA makes use of the connectivity structure from transcriptional regulatory networks and a set of gene expression data to infer dynamics of transcription factor activities. NCA has been successfully applied in inferring a transcriptional regulatory network of the cytokinesis-related genes [11] and phase-specific control elements of its cell cycle in yeast [12]. In this study, we built an integrated dynamic model of the human lung cancer in response to MGd, which consisted of the calculated transcription factor activities, transcription factor regulatory influences on each gene.

Given the complex nature of biological systems, more than one pathway may be involved in any given complex disease. Two or several pathways may interact with each other to cause the disease. This is very likely because functional important proteins may be involved in multiple pathways [13]. Therefore, besides the identification of specific pathways, we also take a further step by exploring the interaction and crosstalk between pathways that related to MGd-treated lung cancer. In this study, we used a computational approach to detect crosstalk among pathways based on a protein-protein interaction (PPI) network, the co-expressed significance of each gene pair, and a scoring scheme which is used to define a function [14].

We defined the dynamic regulated network using NCA which requires two inputs: a set of gene expression profiles and a pre-defined matrix containing the influence of each transcription factor on its estimated or identified target genes. Two outputs of NCA (predicted factor activities and regulatory influences) have added additional insights to gene expression data where the underlying regulatory network structure is partially known. In order to interpret transcription factor activities and regulation strength(influences), the correction between TF activities and expression, hierarchical clustering were calculated. Finally, the dynamic regulated networks were constructed. Beside, PCA was used to detect the relationship among pathways.

In brief, our study aims to reveal molecular mechanism of MGd-treated human lung cancer cells from a dynamic and systematic perspective by PCA and NCA. Our results should provide new avenues for more advanced investigation into the biological role of TFs and pathways in MGd-treated human lung cancer cells.

## Methods

Human lung cancer (A549) cells [15] were treated with 50 uM metal cation-containing chemotherapeutic drug motexafin gadolinium (MGd) for 4, 12, or 24 hours. Their expression profiles were compared with those of the control cells treated by 5% mannitol with the same incubation time. The detail of the samples was shown in Table 1. The limma method [16] was used to identify differentially expressed genes (DEGs) in the expression profile (GSE2189). The DEGs with fold change >1.5 and p-value <0.05 were selected for further analysis. Each selected DEG must be differently expressed in more than one stage. In addition, 6328 regulatory relationships between 250 TFs and 2255 target genes were collected from TRED [17] and TRANSFAC [18].

**Table 1:** The description of samples in GSE2189.

Samples	Samplereplicates	Treated time (hr)	Treatment
4_Hr_+MGd	3	4	50 uM MGd
12_Hr_+MGd	3	12	50 uM MGd
24_Hr_+MGd	3	24	50 uM MGd
4_Hr_No_MGd	3	4	5% mannitol
12_Hr_No_MGd	3	12	5% mannitol
24_Hr_No_MGd	3	24	5% mannitol

In order to add more regulation relationships between TF and target genes, a total of 250 TFs and 144 DEGs were selected to be hierarchically clustered by hcluster of R language. For each pair of TF and its target gene, only the target gene in the sub-tree of the TF-node with a coefficient larger than 0.8 (threshold |r|>0.8) was selected for NCA.

Finally, 627 TF-target genes regulation relationships (containing the TF-TF interactions) were identified based on 164 TFs and 83 DEGs.

### Network Component Analysis

NCA uses the standard log-linear model to approximate the relationship between levels of TFs activity and that of the target-gene expression by assuming the Hill cooperation between TFs on the promoter region of target genes. Formally,
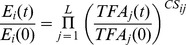
(1)where t represents the time stage, 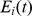 is the gene expression level and 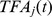 is TF j’s activities and 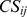 reflects the control strength of TF j on gene i.

Then, the equation (1) is linearized as (in matrix form):
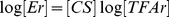
(2)


The matrix 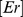 consists of elements 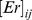 = 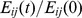, and similarly 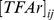 =  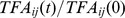 represents the relative gene expression levels and TFs’ activities. The dimension of 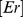 is 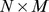
(N genes and M samples or conditions) while that of 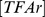 is 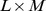
(L TFs). They respectively indicate the time courses of relative gene expression levels and TFs’ activities. Finally, the size of 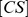 is 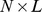, which is the control strength for L TFs on each of N genes. The equation (2) above can be further simplified as:
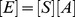
(3)


Here, we have the strength matrix,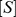, which corresponds to the term of 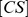 in equation (2) and the TFs’ activity matrix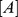, which is the equivalent of 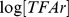 in the equation (2), and finally, the gene expression matrix of 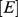 corresponding to the term of 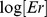 in equation (2).

Based on the above preparation, the decomposition of 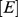 into 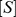 and 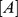 can be achieved by minimizing the following object function:
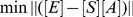
(4)


Subject to. 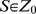


In NCA, the above target function is estimated by using the bootstrap algorithm and the value of 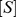 and 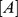 can be normalized through a nonsingular matrix of 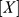 according to
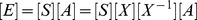
(5)


Specifically, to guarantee uniqueness of the solution for the matrix decomposition of Eq. 4, the network topology needs to satisfy some criteria [19]: (i) the connectivity matrix [A] must have full-column rank. (ii) When a node in the regulatory layer is removed along with all of the output nodes connected to it, the resulting network must be characterized by a connectivity matrix that still has full-column rank. (iii) [P] must have full row rank.

The NCA algorithm implemented in MATLAB by the authors is downloadable at http://www.seas.ucla.edu/~liaoj/. In this study, we followed the manual of this package.

### Pathway Enrichment Analysis

Pathway information was collected from KEGG (Kyoto Encyclopedia of Genes and Genomes), a collection of online databases dealing with genomes, enzymatic pathways, and biological chemicals [20]. DAVID [21], a high-throughput and integrated data-mining environment, was utilized to analyze gene lists derived from high-throughput genomic experiments. Significant pathways that have at least two members and p-value <0.1 were thus selected.

### Pathway Crosstalk Analysis

PPI data were collected from the HPRD [22] and BIOGRID [23]. A total of 326119 unique PPI pairs were collected to construct the PPI network. Limma eBayes method [16] was used to measure the differential expression status of genes. Pearson correlated coefficient test was employed to determine the co-expressed significance of each gene pair. The above two types of values were mapped to the nodes and edges in the PPI network. The following formula was used to define a function as the combination of statistical significance of an interaction by a scoring scheme [24]. The detail could be seen in Liu et al [14].
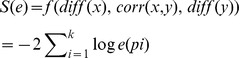



The diff(x) and diff(y) are differential expression assessments of gene x and gene y, respectively. Corr(x' y) represents the correlation between gene x and gene y. f is a general data integration method that can handle multiple data sources differing in statistical power. Where k = 3, p1 and p2 are the p-values of differential expression of two nodes, and p3 is the p-value of their co-expression.

To define the interaction significance between pathways, we summarized all the scores of edges S(e) of all non-empty overlaps. Specifically, the interaction score between two pathways was estimated according to their overlapping status of weighted pathways in the following formula:
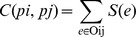
where Pi and Pj are two pathways and Oij is their overlapping. To estimate the significance of the overlapping between different pathways, we randomly sampled 1×10^6^ times of the two same size pathways in the edges of pathway network and calculated their overlapping scores. The frequency larger than a C score denoted significant interaction between Pi and Pj.

## Results and Discussion

### Transcription Factor Activities

**Figure 1:**
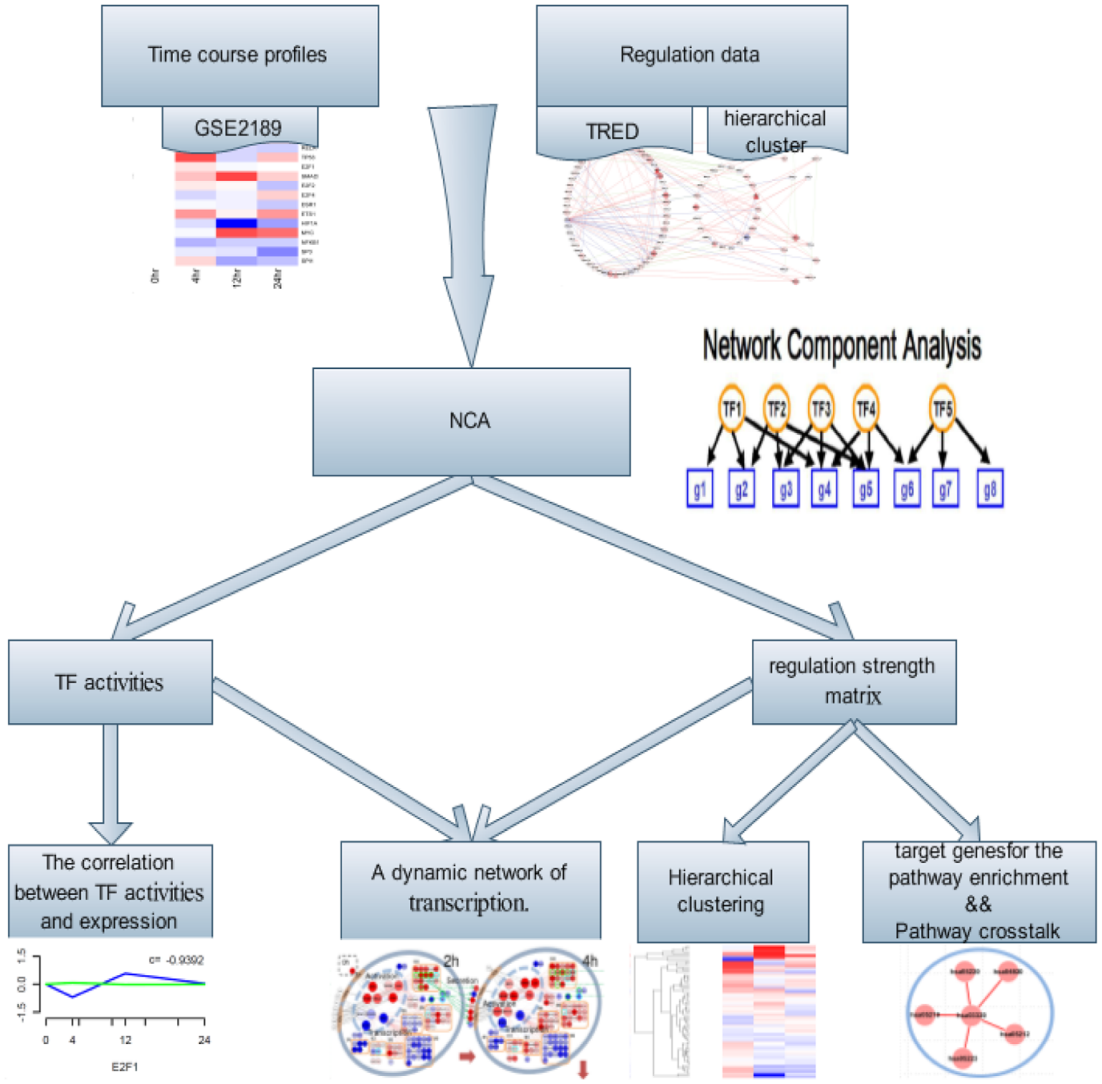
Flowchart of dynamic transcriptional regulatory network construction.

**Figure 2:**
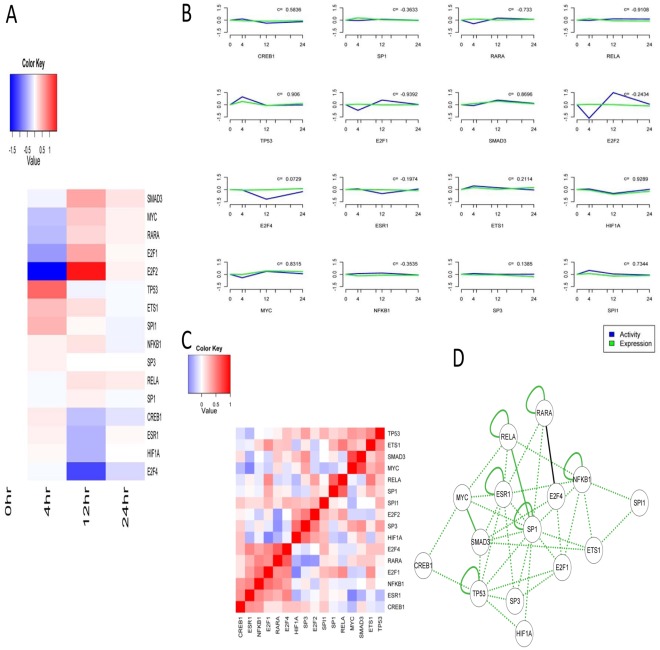
Transcription factor activities calculated using NCA. (A) Predicted activities of the 16 TFs used in this study. Rows represented progression in time and columns corresponded to the activities. Activities of each column were normalized to the zero time point. (B) Transcription factor activities (blue) compared with gene expression (green), with Pearson correlation coefficients noted. Both activity and expression at each time point were averages normalized to the time zero values. (C) Correlation matrix between transcription factor activities. Red represented positive correlation and blue represented negative correlation. (D) Inferred combinatorial regulation pairs of TFs. Green solid line indicated that the pair was supported by protein-protein interaction information from HPRD, BIOGRID and high correlation of their activities (>0.6). Black solid line indicated that the pair was only supported by high correlation, and a green dotted line indicated that the pair was only supported by the interaction database.

The schematic of our approach was shown in Figure 1. Based on NCA method, 16 TFs were screened to construct a dynamic regulatory network. Figure 2A and 2B showed the estimated activities of the 16 TFs. Transcription factor activities clearly showed early-, mid-, and late-phase action in response to MGd. SP1, RARA, RELA, TP53, ETS1, and SMAD3 were activated within 4 hours after the MGd was injected. SP1 activation peaked at 4 hours and HIF1A, CREB1 and SPI1 were predicted to be somewhat deactivated over 12 hours (Figure 2A). Research found that Sp1 level accumulated strongly in early stage and then declined in late stage [25] and Aryl hydrocarbon receptor in association with RelA modulates IL-6 expression in non-smoking lung cancer [26]. These are evidence which could improve the reliable of research.

The calculated transcription factor activities were compared with the gene expression data for each transcription factor (Figure 2B).TP53, SMAD3, and HIF1A showed strong positive correlation between activities and expression (correlation coefficient r>0.8) (Figure 2B). However, transcription factor activities were sometimes, but not always, correlated with the gene expression of the TFs.

We also compared the significant correlation between transcription factor activities with published protein-protein interactions catalogued in the HPRD [22] and BIOGRID [23]. Interestingly, TFs known to act together showed high correlation in their activity profiles (Figure 2C). For example, the highly correlated TFs SP1 and RELA regulated their target genes together [27].

Our results also revealed several interactions between TFs (Figure 2D). The transcription factor Sp1 regulates expression of numerous genes involved in various cellular processes, and dysregulation of Sp1 is observed in many cancers and diseases [28]. Involvement of ESR1 in lung cancer has also been observed [29]. Interaction of SP1 with ESR1 has been demonstrated in breast cancer cells [30]. In addition, E2F1 and SP1 participate in cell proliferation and viability via regulating phosphocholine cytidylyltransferase alpha (CCTα) [31]. Thus the predicted ESR1/Sp1 and E2F1/Sp1 interactions may suggest their regulation role in the pathogenic process. Estrogen stimulation can enhance the c-MYC-ESR1 interaction and facilitate the association of ESR1, c-MYC, and the co-activator TRRAP with these estrogen-responsive promoters, resulting in chromatin remodeling and transcription increase in breast cancer. These suggest ESR1 and c-MYC may physically interact to stabilize the ESR1-coactivator complex, thereby permitting other signal transduction pathways to fine-tune estrogen-mediated signaling networks [32]. C-Myc, an oncogene, has also been demonstrated to interact specifically with Smad3, one of the signal transducers involved in TGF-β signaling which is involved in cancer development [33]. As for NFKB1/RELA, NFKB1 or NFKB2 could bind to RELA, RELB or REL to form the NFKB family of TFs. These hetero-dimers participate in controlling a wide variety of genes, and are important in embryonic development, apoptosis, immune, inflammatory and stress responses. The NFKB1/RELA complex is the most abundant form of NFKB. In HeLa cells, RELA phosphorylation could result in increased transcription of NFKB target genes and inhibiting apoptosis [34].

### Significant Regulation Relationships between TFs and Target Genes

**Figure 3:**
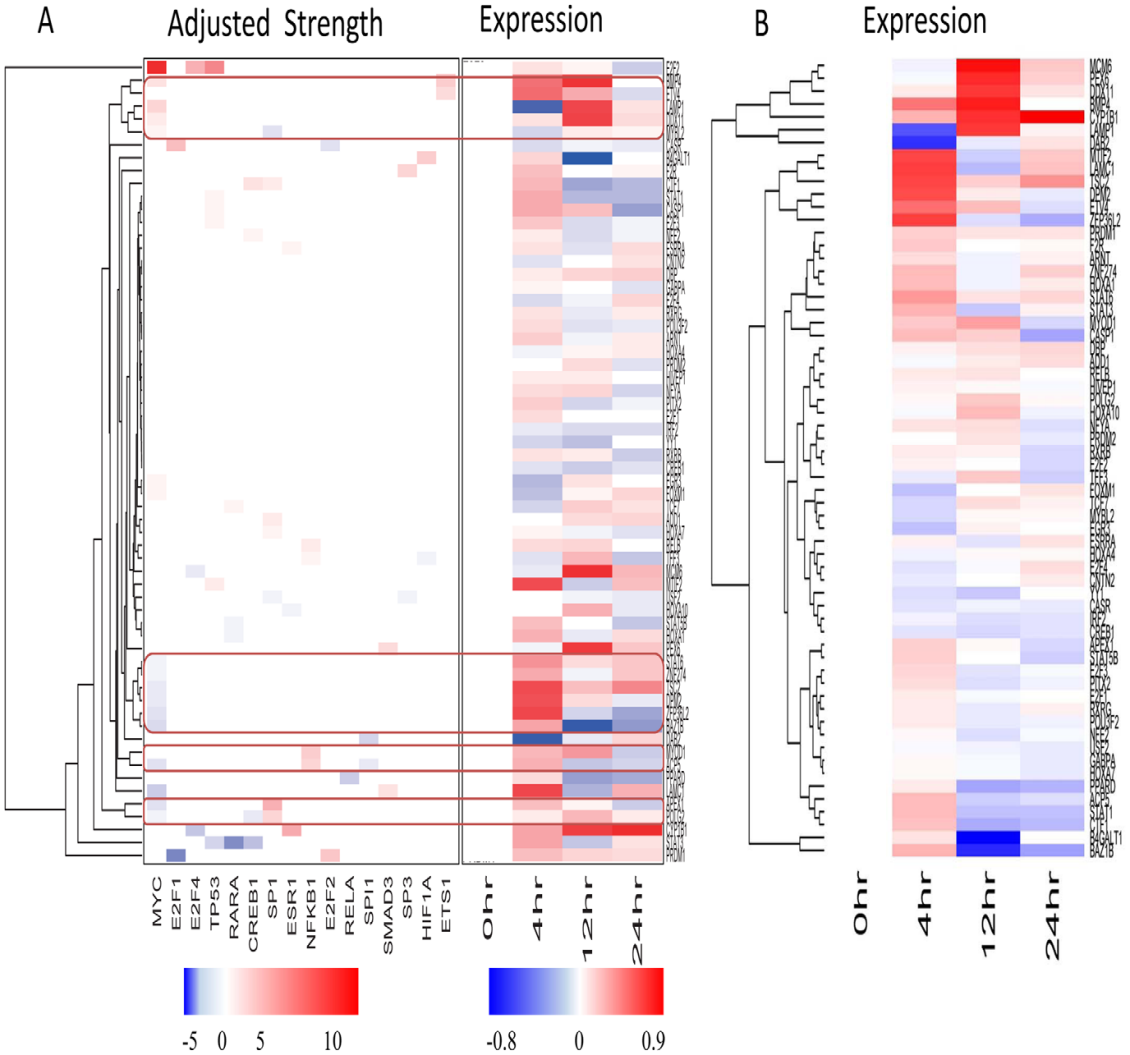
Hierarchical clustering in the context of a defined regulatory network. (A) The adjusted strength matrix was used for clustering, with the gene expression matrix appended. Four major clusters, which have more than three associated genes, were highlighted. In the adjusted strength matrix heatmap, white indicated a weak regulatory influence. (B) Clustering with gene expression.

In Figure 3A, target genes were hierarchically clustered with the adjusted strengths of TFs and shown with gene expression. We identified several major clusters, which were correlated to the coordinated action of TFs to regulate gene expression. We found that MYBL2, DDX11, LAMP1, ETV4, and BMP4 were regulated by MYC and ETS1. The MYC independently regulated BAZ1B, ZFP36L2, DPM2, TSC2, ZNF274, and STAT6. MYOD1 and ACP5 were regulated by the NFKB1. APEX1 and POLG2 were regulated by SP1 and CREB1. Target genes were hierarchically clustered with the expression of TFs Figure 3B. Some of them in Figure 3B haven’t been emerge in Figure 3A. The clusters shown in Figure 3A suggested that we might be able to use our cluster information to discover new regulatory relationships.

We found five significant regulation relationships which were proved by the previous research: BMP4/Est1, TSC2/myc, APE1/Sp1/p53, RARA/HOXA1, and SP1/USF2. BMP4 signaling induces senescence and modulates the oncogenic phenotype of lung cancer cell [35]. BMP4 promoter has two Ets-1 binding sites, and Ets-1 activity is increased in hepatocellular carcinoma cells under hypoxic conditions. Thus over-expression of Ets-1 markedly enhances BMP4 promoter activity [36]. In addition, BMP4 is associated with Smad and p38 MAPK pathway in lung cancer cell, which was also observed in our regulatory network [28].

MYC could directly affect transcription of tuberous sclerosis 2 (TSC2), as shown by quantitative mRNA analyses and by Myc binding to its promoter in chromatin immunoprecipitation assays. Importantly, myc-null experiments have shown that Myc acts as a strong and direct repressor for TSC2 expression because its loss results in increased TSC2 mRNA. This finding shows that regulation of TSC2 may contribute to the effects of MYC on cell proliferation and neoplastic growth [37], [38].

The putative promoter region of the Apex1 gene contains CCAAT boxes and a CpG island possessing putative binding sites for several TFs, such as Sp1 [39]. The Sp1 site upstream of the transcription start, together with an adjacent CCAAT element, establishes a protein-DNA complex required for basal transcription of APEX1 [40]. Further study indicates that p53 provides a mechanism for the down-regulation of APE1 by interfering with Sp1 binding to the APEX1 promoter. These findings demonstrates that p53 is a negative regulator of APE1 expression in response to DNA damage [41].

RARA is one ligand dependent inducible transcription factor. The RARs family can activate gene expression directly through RA responsive elements (RAREs) localized in their target genes. Functional RAREs are currently known for only a few HOX genes, including HOXA1, HOXB and HOXC [42].

As a member of the bHLH family, USF-2 has been demonstrated to specifically bind with E-box motif A, located between −147 and −142 in the human [Arginine]vasopressin promoter to be involved in small cell lung cancer [43], [44]. However, there was evidence that a physical interaction between USF2 and Sp1/Sp3 [45], suggesting USF-2 may exert important roles in lung cancer through interaction with E-Box or GC box.

### Overall Regulatory Dynamics in Response to MGd

**Figure 4:**
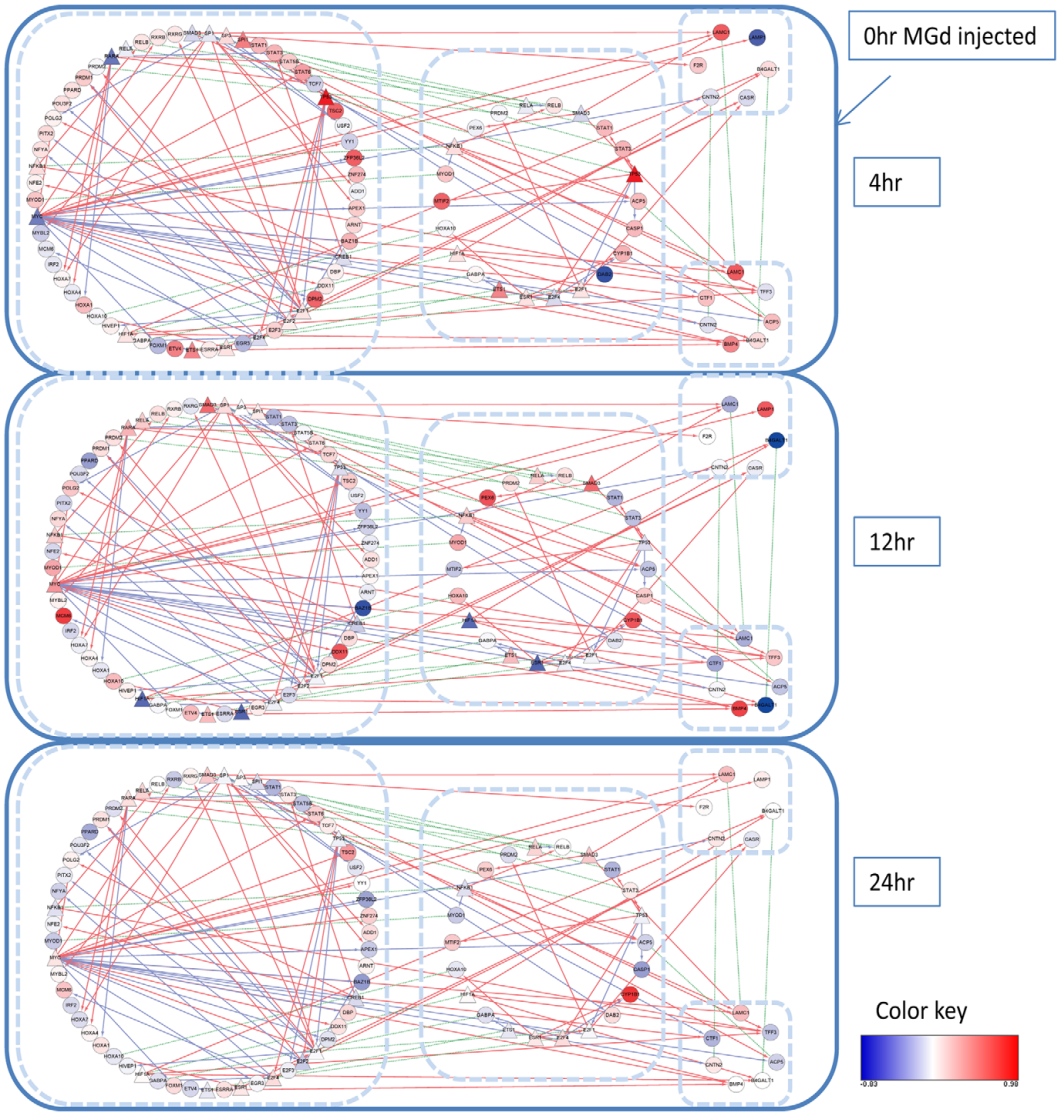
A dynamic network of transcription in response to MGd. Target genes were noted with circles, and TFs with triangle. The 4 subcellular locations (Nucleus; Cytoplasm; Plasma membrane; Extracelluar) were grouped in 4 cycles. Green dotted lines denoted of a target gene which may transfer between two subcellular locations. Red and blue lines showed the influence of a transcription factor on a target gene.

We built an integrated dynamic model of the human lung cancer in response to MGd (Figure 4), which consisted of the calculated transcription factor activities, transcription factor regulatory influences on each gene, subcellular location, and the gene expression data. During the first 4-hour period, TP53, SP1, E2F1, ETS1, SMAD3 and RELA were activated and interacted to regulate gene expression. These TFs had already affected gene expression including the genes in the Nucleus and Cytoplasm after 4 hours. SMAD3, also expressed in the Nucleus and Cytoplasm, showed peak activity at 12 hours and then at 24 hours returned to the previous 4 hours level. By contrast, E2F1 activation rapidly returned to the base level of activity.

### Pathway Crosstalk Analysis

Most of the significant pathways (p-value <0.1 using the hypergeometric test) were cancer related signaling pathways (Table 2), including Pathways in cancer, Small cell lung cancer, Non-small cell lung cancer, Pancreatic cancer, Jak-STAT signaling pathway, PPAR signaling pathway and so on. These pathways have been demonstrated involved in lung cancer in previous works. For example, BMP4 treatment (enriched in the Pathways in cancer) has been suggested to induce a senescent morphology in A549 lung cancer cells [35]. Unphosphorylated signal transducers and activators of STAT6 (enriched in the Jak-STAT signaling pathway) may transcriptionally up-regulate cyclooxygenase-2 expression and protect against apoptosis in NSCLC cells [46]. PPARγ has been suggested to modulate the proliferation and apoptosis of lung cancer cell through interaction with its ligand. PPARγ expression is found higher in lung cancer cell patients when compared with normal surrounding tissue. The treatment of lung adenocarcinoma cells (A549) with troglitazone (a PPARγ ligand) can enhance PPARγ transcriptional activity and induce a dose-dependent inhibition of A549 cell growth [47], [48], [49]. In brief, activation of PPARγ impedes lung tumor progression and PPARγ ligands may serve as potential therapeutic agents for lung cancer. In this study, we found PPAR signaling pathway was an important pathway in response to MGd-treatment, suggesting MGd may be one potential PPARγ ligand as troglitazone.

**Table 2:** Significant pathways.

Pathway ID	Description	P-value
hsa05200	Pathways in cancer	2.97E-07
hsa05222	Small cell lung cancer	1.55E-04
hsa05223	Non-small cell lung cancer	3.28E-04
hsa05212	Pancreatic cancer	9.85E-04
hsa05215	Prostate cancer	0.002168
hsa05221	Acute myeloid leukemia	0.005524
hsa04110	Cell cycle	0.007346
hsa05220	Chronic myeloid leukemia	0.011247
hsa05216	Thyroid cancer	0.014013
hsa04630	Jak-STAT signaling pathway	0.015367
hsa05219	Bladder cancer	0.028249
hsa05214	Glioma	0.059075
hsa04920	Adipocytokine signaling pathway	0.065857
hsa03320	PPAR signaling pathway	0.069342
hsa05218	Melanoma	0.072888

**Figure 5:**
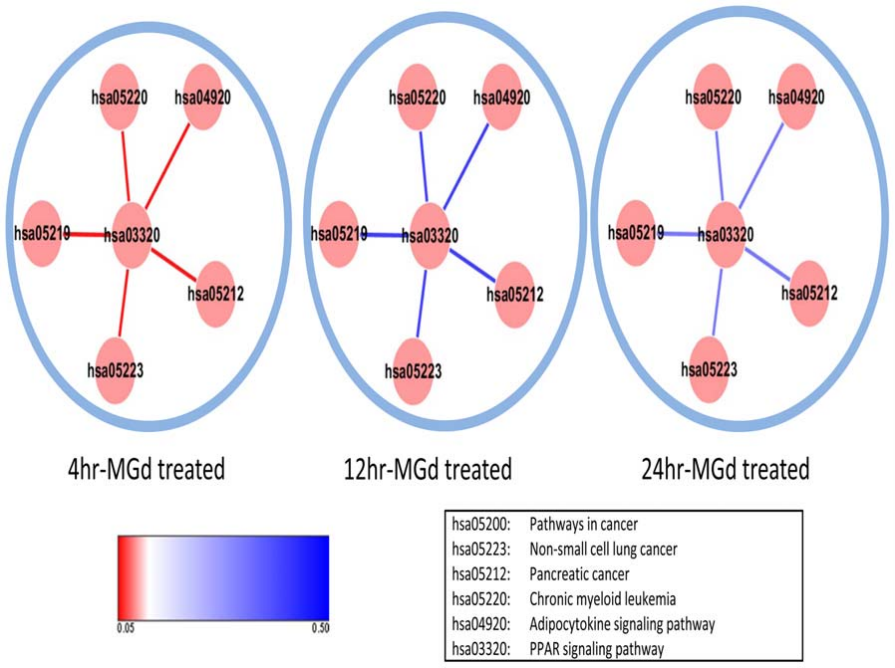
Dynamic of pathway-crosstalk. The red line indicated the p-value of cross-talking between two pathways less than 0.1. The blue line indicated the cross-talking was not significant with the p-value large than 0.3.

We further analyzed the pathway’s interactions and calculated a C score of each pair of pathways. PPAR signaling pathway (hsa03320) was found cross-talking with the pathway of Non-small cell lung cancer (hsa05223; p-value = 0.056135), Pancreatic cancer (hsa05212; p-value = 0.056145), Bladder cancer (hsa05219; p-value = 0.056165), Adipocytokine signaling pathway (hsa04920; p-value = 0.056214), and Chronic myeloid leukemia (hsa05220; -value = 0.056214) after 4-hour MGd treatment (Figure 5). But this crosstalking was not significant at the 12-hour and 24-hour stages with the p-value >0.1.

Overall, we suggest PPAR signaling pathway (hsa03320) plays an important role in the pathways crosstalk.

There was evident of interaction relationship between PPAR signaling pathway and Adipocytokines signaling pathway in previous study [50], [51]. Among them, adipocytokines, secreted from adipocytes, such as tumor necrosis factor-alpha (TNF-a), plasminogen activator inhibitor type 1 (PAI-1), interleukin 6 (IL-6), leptin, resistin, and adiponectin, play a significant role in normal metabolic homeostasis and in the development of several diseases. Leptin could be decreased regulation by PPAR-γ agonists. PPAR-γ and liver receptor homolog-1 (LRH-1) play significant roles in adiponectin transcriptional activation by means of PPRE and LRH-RE in its promoter [50], [51].

However, there are still some limitations in our research. Our study is based on an assumption that mRNA expression levels are controlled entirely by transcriptional regulation. However, mRNA stability is also a less informative factor to the mRNA expression levels [52], [53]. If an mRNA whose expression level is determined by mRNA stability can also express in the control cells, we can exclude the influence of mRNA stability by comparison when their degradation rates are similar. If such an mRNA cannot express in the control cells, we ignore the mRNA stability. This may bring systematic errors to this study. In addition, the quality and quantity of Protein-protein interaction (PPI) data is one of the problems for the PCA. PCA was based on a PPI interaction data [24]. Protein-protein interactions provide valuable information about how genes carry out their biological functions. It is expected that protein-protein interaction data information will be widely accessible in the near future by using various experiment methods.

### Conclusion

We managed to interpret the molecular mechanism of lung cancer from a systematic and dynamic perspective by NCA. We took the control strength (only as positive or negative) as the regulatory relationships between TFs and their target genes (including TFs), and the TFs activities was substituted for their gene expression to construct the dynamic network. Using NCA, the significant TFs and their target genes were detected, the control strength of TFs to their target genes was recalculated, and the activities of the TFs were estimated.

NCA and PCA methods were applied to explore the transcription response mechanism in MGd-treated human lung cancer cells based on the assumption that lung cancer is a contextual attribute of distinct patterns of interactions between multiple elements. The results identified a set of key TFs, target genes for these TFs and signaling pathways involved in regulatory networks. Through the activity of TFs, we found that transcription factor activities clearly showed early-, mid-, and late-phase action in response to MGd. We also identified several major clusters, which were correlated to the coordinated action of TFs to regulate gene expression. Besides, pathway-crosstalk analysis indicated there was an interaction relationship between PPAR signaling pathway and Adipocytokines signaling pathway in our study. Finally, an integrated dynamic model of the human lung cancer was built in response to MGd (Figure 4), which consisted of the calculated transcription factor activities, transcription factor regulatory influences on each gene, subcellular location, and the gene expression data.

The development of new high-throughput technologies greatly produces great amounts of biology data. Then how to mine meaning information from the data become necessary. Our studies revealed that NCA and PCA could be successfully applied for inferring the transcriptional regulatory network of MGd-treated human lung cancer.
